# Feasibility of Non-contrast-enhanced MR Angiography Using the Time-SLIP Technique for the Assessment of Pulmonary Arteriovenous Malformation

**DOI:** 10.2463/mrms.mp.2015-0069

**Published:** 2016-02-03

**Authors:** Kohei HAMAMOTO, Katsuhiko MATSUURA, Emiko CHIBA, Tomohisa OKOCHI, Keisuke TANNO, Osamu TANAKA

**Affiliations:** Department of Radiology, Jichi Medical University, Saitama Medical Center 1-847 Amanuma-cho, Omiya-ku, Saitama 330-8503, Japan

**Keywords:** pulmonary arteriovenous malformations, time-spatial labeling inversion pulse (time-SLIP), non-contrast-enhanced magnetic resonance angiography, fast advanced spin echo (FASE), coil embolization

## Abstract

**Purpose:**

The purpose of this study was to evaluate the diagnostic performance of non-contrast-enhanced magnetic resonance angiography with time-spatial labeling inversion pulse (time-SLIP MRA) in the assessment of pulmonary arteriovenous malformation (PAVM).

**Methods:**

Eleven consecutive patients with 38 documented PAVMs underwent time-SLIP MRA with a 3-tesla unit. Eight patients with 25 lesions were examined twice, once before and once after embolotherapy. The lesions were divided into two groups—initial diagnosis (n = 35) and follow-up (n = 28)—corresponding to untreated and treated lesions, respectively, and were evaluated separately. To evaluate the initial diagnosis group, two reviewers assessed image quality for visualization of PAVMs by using a qualitative 4-point scale (1 = not assessable to 4 = excellent). The location and classification of PAVMs were also evaluated. The results were compared with those from digital subtraction angiography. For evaluation of the follow-up group, the reviewers assessed the status of treated PAVMs. Reperfusion and occlusion were defined respectively as visualization or disappearance of the aneurysmal sac. The diagnostic accuracy of time-SLIP MRA was assessed and compared with standard reference images. Interobserver agreement was evaluated with the κ statistic.

**Results:**

In the initial diagnosis group, time-SLIP MRA correctly determined the PAVMs in all but one patient with one lesion who had image degradation due to irregular breath. Image quality was considered excellent (median = 4) and the κ coefficient was 0.85. Additionally, both readers could correctly localize and classify the PAVMs on time-SLIP MRA images with both κ coefficient of 1.00. In the follow-up group, the sensitivity and specificity of time-SLIP MRA for reperfusion of PAVMs were both 100%, and the κ coefficient was 1.00.

**Conclusion:**

Time-SLIP MRA is technically and clinically feasible and represents a promising technique for noninvasive pre- and post-treatment assessment of PAVMs.

## Introduction

Pulmonary arteriovenous malformation (PAVM) represents a direct connection between the pulmonary arterial circulation and the pulmonary venous circulation via a fragile, thin-walled aneurysmal sac.^1,2^ PAVM is strongly associated with hereditary hemorrhagic telangiectasia (HHT), and is present in up to 30% of HHT patients.^3,4^ Patients with PAVM may be at risk for hemoptysis and serious neurologic complications, including migraine, transient ischemic attack, stroke, cerebral abscess, and seizure.^5–7^ Endovascular treatment consisting of embolization of the arterial side of the malformation with metallic coils has been accepted as the standard of care.^1,8,9^ It is crucial to assess PAVM patency before and, equally important, after embolization in order to detect reperfusion of the lesion, which occurs in up to 50% of cases.^10–15^ Digital subtraction angiography (DSA) is commonly performed to evaluate PAVM patency because it can provide simultaneous hemodynamic and morphological information. However, DSA is invasive, and the risk from radiation exposure limits its routine use in the setting of diagnosis and follow-up. In recent years, multi-detector computed tomography (MDCT) with contrast has become important in the evaluation of PAVMs because it is noninvasive, has high spatial resolution, and requires shorter examination times.^12,13,15,16^ However, as with DSA, MDCT necessitates exposure to ionizing radiation and to risks associated with potentially nephrotoxic iodinated contrast agents. These risks may be of particular concern for young patients and women of child-bearing age who require regular follow-up over an extended period, as well as in patients with renal insufficiency. Furthermore, MDCT provides only an indirect assessment of PAVM patency via evaluation of the size of the artery, vein, and aneurysmal sac, which is another drawback of this method.

Contrast-enhanced magnetic resonance angiography (MRA) has now been shown to be a useful method for evaluation of PAVM patency.^17,18^ More recently, with advances in MR imaging, dynamic MRA, also known as time-resolved MRA, has become available and may be useful to diagnose PAVMs while maintaining both adequate spatial and temporal resolutions.^19,20^ Although dynamic MRA is now regarded as an acceptable modality for noninvasive assessment of PAVM patency, concerns about serious adverse reactions, including anaphylaxis and nephrogenic systemic fibrosis,^21^ to gadolinium contrast agents, remain a major drawback.

Non-contrast enhanced MRA with time-spatial labeling inversion pulse technique (time-SLIP MRA) is a relatively new MRA protocol that allows selective visualization of blood flow in a region of interest using an arterial spin labeling technique. Time-SLIP MRA can provide both morphological and hemodynamic information without ionizing radiation and without administration of contrast material.^22^ To date, this technique has been successfully applied in evaluation of the portal vein^23,24^ and of cerebrospinal fluid^25^ and dural arteriovenous fistulas,^26^ and Ohno et al. have recently reported the usefulness of time-SLIP MRA for pre-surgical anatomical assessment of pulmonary arteries or veins in patients with non-small cell lung carcinoma.^27^ However, to our knowledge, there have been no reports concerning clinical application of non-contrast-enhanced MRA for the assessment of PAVMs. The purpose of this study was to assess the feasibility of non-contrast-enhanced time-SLIP MRA for the initial diagnosis and post-treatment follow-up of PAVMs.

## Materials and Methods

### Patients

The prospective study design was approved by our ethics committee. We identified 12 consecutive patients who were scheduled to undergo endovascular treatment of PAVMs between October 2012 and December 2014. One patient who had persistent arterial fibrillation was excluded because the time-SLIP MRA protocol for this study required electrocardiography gating, and it was expected that severe image degradation would occur in patients with chronic arrhythmias. Therefore, this study included 11 patients, two men and nine women aged 22 to 81 years (median, 53 years). All patients were informed of the details of this study and provided written consent for the use of their medical records for research purposes. Five patients had been diagnosed with hereditary hemorrhagic telangiectasia (HHT). Nine of the 11 patients had newly diagnosed PAVMs and the other 2 were scheduled to undergo embolotherapy for residual untreated lesions. There were 42 PAVMs in the 11 patients (range, 1 to 22), among which five lesions in 2 patients had previously been treated by embolization with 0.010 to 0.014-inch metallic coils. All patients underwent thin-section contrast-enhanced CT before time-SLIP MRA for evaluation of the patency and angiostructure of the PAVMs. Four lesions (2 untreated and 2 treated) in two patients could not be evaluated by time-SLIP MRA due to limited acquisition volume. Therefore, a total of 38 lesions (35 untreated and 3 treated) were analyzed by initial time-SLIP MRA. Definitive diagnosis of PAVM was made within 1 or 2 days after image acquisition by time-SLIP MRA. After the initial evaluation, 27 of the 35 untreated lesions were embolized using 0.010- to 0.014-inch platinum coils and 3 treated lesions were also re-embolized with platinum coils because of recanalization. One patient underwent embolotherapy twice, and one patient underwent embolotherapy thrice, because of multiple lesions in both lung fields. Eight patients with 25 treated lesions were available for follow-up time-SLIP MRA evaluation. One patient with one lesion was withdrawn from follow-up and another patient with one lesion chose not to undergo the follow-up MRA because of the long acquisition time. The other patient with three lesions did not undergo follow-up study yet when this study was concluded. Of the eight remaining patients who were available for follow-up time-SLIP MRA, those who underwent multiple embolizations (n = 2) underwent a corresponding number of follow-up MRA studies (n = 2 studies in one patient and 3 studies in the other). All eight patients also underwent time-resolved contrast-enhanced MRA and contrast enhanced CT at various intervals for evaluation of PAVM patency after embolotherapy. Follow-up time-SLIP MRA was performed at the same time as time resolved contrast-enhanced MRA. Images acquired by initial and follow-up time-SLIP MRA were divided into two groups and analyzed separately based on criteria for image assessment described in the following section. Group 1 (initial diagnosis) consisted of time-SLIP MRAs performed for the initial diagnosis of previously unknown PAVMs. Group 2 (follow-up) included all evaluations that were performed after endovascular treatment of PAVMs. Finally, the initial diagnosis and follow-up groups included 35 untreated lesions and 28 treated lesions, respectively.

### Time-SLIP MRA protocol

**Fig. 1.**
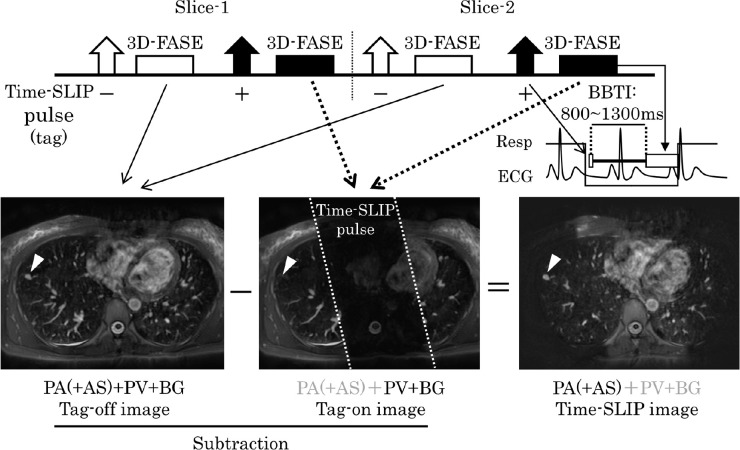
Procedure for acquiring non-contrast-enhanced magnetic resonance angiography with time-spatial labeling inversion pulse (time-SLIP MRA). Three-dimensional (3D) single-shot half-Fourier fast spin echo (FSE) (fast advanced spin echo: FASE) images with and without time-SLIP (tag) pulse were acquired alternately in the same slice plane. The tag-off image shows the signals of pulmonary vascular structures including pulmonary artery (PA), pulmonary vein (PV), aneurysmal sac (AS), and background (BG). Time-SLIP pulse was applied to the right-sided blood flow. Because the blood from the tagged region showed a decrease in signal, the signals of PA and AS were suppressed on the tag-on image. Consequently, these images were subtracted, and as a result, only signals of the PA and AS were described on time-SLIP. The arrowheads indicate the aneurysmal sac. Note that the signal of the aneurysmal sac is attenuated on the tag-on image. BBTI, black blood inversion time; ECG, electrocardiography-triggering; Resp, respiratory-triggering

All MRAs were performed with a 3-tesla MRI system (Vantage Titan 3T, Toshiba Medical Systems, Tochigi, Japan) using four- and eight-element phased array body surface coil and receiver channels combined with parallel imaging capability (Speeder, Toshiba Medical Systems). To localize the heart for placement of time-SLIP pulse (tag) and to determine the acquisition area, axial, coronal, and sagittal field echo images without an inversion pulse were acquired using the following parameters: repetition time/echo time (TR/TE), 50/2.3; flip angle, 30°; field of view, 400 × 400 mm; matrix, 256 × 128; number of slices, 7; and slice thickness, 10 mm. To visualize the pulmonary vessels selectively, combined respiratory- and electrocardiography (ECG)-triggered three-dimensional (3D) single-shot half-Fourier fast spin echo (fast advanced spin echo: FASE, Toshiba Medical Systems) images with fat saturation were acquired in the axial plane using time-SLIP with an alternate tag-on/off subtraction technique. Details of the procedures of this time-SLIP technique were described previously.^22^ Briefly, 3D FASE images with and without time-SLIP pulse (tag) were acquired alternately in the same slice plane, generating two separate images. When a tag is used as the labeling slab, the blood from the tagged region shows a decreased signal. These images are individually reconstructed and then subtracted to yield the final angiogram. Consequently, this technique allows depiction of only signals from the tagged region, which is referred to as the time-SLIP image, by cancellation of the background signal via subtraction. Figure 1 shows the procedures for image acquisition and reconstruction. The 3D FASE scan was conducted with the following parameters: 1 respiration interval/1; flip angle, 90°; echo-train spacing, 30 ms; slice thickness, 2.0 mm; inversion time, 180 ms for fat suppression; slice section, 60 for tag-on and tag-off images, respectively; field of view, 370 × 370 mm; matrix size, 256 × 256; reconstructed matrix size, 512 × 512; number of acquisitions, 1; and a parallel imaging factor of 2 in the phase direction. The final images were reconstructed into apparent spatial resolutions of 1 × 0.6 × 0.6 mm. The total scanning times were approximately 20 minutes. Due to a limitation in the acquisition volume of time-SLIP MRA, we set the position of each slab to include the feeding artery, draining vein, and aneurysmal sac along with portions of the main pulmonary artery and vein of the target PAVMs by referring to recent contrast-enhanced CT images. Respiratory triggering was conducted at the beginning of expiration. In ECG gating, the trigger time of both time-SLIP pulse and 3D FASE image acquisition was set at diastole. The interval between time-SLIP pulse application and 3D FASE image acquisition was within one cardiac cycle. A time-SLIP pulse (tag) was placed on the range that covered the right atrium and ventricle and the superior and inferior vena cava in the sagittal direction. The delay after the time-SLIP pulse, which is referred to as the black blood inversion time (BBTI), was 800 to 1300 ms, due to variations of the R-R intervals of each patient. Before the current study, the appropriate BBTI described above was determined by the same sequence for BBTIs ranging from 400 to 1500 ms in steps of 100 ms, and was empirically defined for best visualization of pulmonary vessels. This finding was obtained from our own institutional experience (unpublished data).

### Reference standard imaging

DSA was used as the reference standard for all 35 lesions in the initial diagnosis group and for 24 of 28 lesions in the follow-up group. In cases without available DSA due to clinical circumstances (4 lesions in 3 patients), time-resolved contrast-enhanced MRA and contrast-enhanced CT was used instead. All DSA examinations were performed within 48 h after time-SLIP MRA. DSA was performed immediately before embolotherapy with an image intensifier fluoroscopy system (DFP-2000A; Toshiba Medical Systems) using a femoral vein approach. A 5-F pre-shaped diagnostic catheter (Goodman Co., Ltd, Aichi, Japan) was guided into a subsegmental pulmonary artery that was connected to the targeted PAVM via an introducer sheath. A 2.5-F microcatheter (Renegade-18 Fiber Braided Microcatheter, Boston Scientific Japan, Tokyo, Japan) was introduced into the 5-F catheter and the feeding artery was selectively catheterized. Thereafter, patency and angiostructure were confirmed by manual injection of iodinated (300 mgI/mL) contrast material (Omnipaque 300, Daiichi Sankyo Co. Ltd., Tokyo, Japan).

Time-resolved contrast-enhanced MRA was performed using a 3D fast field-echo sequence in conjunction with a parallel imaging technique and a segmented k-space sampling technique.^28^ All studies were performed on the 3.0-tesla MR system using a four- and eight-element body surface coil. Imaging parameters were as follows: TR/TE, 3.7/1.3; flip angle, 20°; matrix, 256 × 160; reconstruction matrix, 512 × 320; FOV, 370 mm × 370 mm; section thickness, 1.5 mm; number of excitations, 1; and parallel imaging factor, 2, which was acquired with a 3D fast-field echo sequence combined with a sensitivity-encoding technique. For time-resolved contrast-enhanced MRA, a 120- to 132-mm thick 3D slab with 40 to 44 partitions was used. The acquired slice sections of 3 mm were reconstructed with 1.5-mm voxel intervals by means of mid-slice reconstruction. The voxel size of time-resolved contrast-enhanced MRA for all patients was 1.2 × 0.9 × 0.7 mm^3^ or larger. Due to the limitation in the acquisition volume of time-resolved contrast-enhanced MRA as time-SLIP MRA, we set the position of each slab to include the target PAVMs by referring to recent contrast-enhanced CT images. The temporal resolution for each 3D dataset in time-resolved contrast-enhanced MRA was 5.0 seconds, and six dynamic scans were obtained during 30 seconds of breath-holding at end-inspiration. Data acquisition began simultaneously with injection of 0.1 mmol/kg of gadopentetate dimeglumine (Magnevist, Bayer HealthCare, Whippany, NJ, USA) or gadoteridol (ProHance, Eisai Co. Ltd., Tokyo, Japan) at a flow rate of 2 mL/s, followed by a saline flush of 20 mL during breath holding. All source images from each frame were reconstructed with a maximum intensity projection (MIP) algorithm.

Chest computed tomography (CT) scanning was performed using a 64-detector row scanner (Aquilion 64, Toshiba Medical Systems) with a collimation of 64 × 0.5 mm and a pitch of 0.83 or a 320-detector row scanner (Aquilion ONE, Toshiba Medical Systems) with a collimation of 80 × 0.5 mm and a pitch of 0.83. The scanner settings were 120 kV with the tube current set automatically by the exposure control system. Images were acquired during inspiration. After non-contrast CT scanning, a total of 80 to 100 mL iodinated contrast material was administered intravenously at a rate of 3 to 5 mL/s, and CT images were acquired at 7 and 16 s after initiation of contrast administration. All scans were reconstructed in transverse orientations at a slice thickness of 0.5 mm and increments of 0.5 mm with both mediastinal and lung windows. The sizes of feeding arteries and aneurysmal sac were measured on the lung windows.

### Image assessment

The time-SLIP MRA, time-resolved contrast-enhanced MRA, and contrast-enhanced CT data were transferred to an independent workstation (Ziostation 2, Ziosoft, Tokyo, Japan) equipped with multi-planar reconstruction (MPR) for source images and processed as MIP images in three orthogonal orientations. Images were independently analyzed by two experienced radiologists. Both observers viewed sets of images, including all examinations, in a random fashion to reduce recall bias. Disagreements between the reviewers were resolved by discussion. For evaluation of the images in the initial diagnosis group, the reviewers were aware that all examinations were performed in patients with PAVMs, but they were blinded to the location and type of PAVMs and to the results of the DSA, time-resolved contrast-enhanced MRA, and contrast-enhanced CT examinations. First, the examiners assessed the technical feasibility of evaluation of PAVM by time-SLIP MRA. Image quality for the visualization of PAVMs was scored with a four-point scale (1 = severely limited, no visible aneurysmal sacs and feeding arteries; 2 = fair, visualization of suspected aneurysmal sacs and feeding arteries was possible but the continuities were not clear; 3 = good, confident findings of aneurysmal sacs and feeding arteries and their continuities; 4 = excellent, sharply defined vascular structures). Images with scores 3 and 4 were considered as a positive diagnosis of PAVM. Next, the reviewers assessed the segmental location of the aneurysmal sac and classification of PAVMs in the cases scored 3 and 4 using criteria adopted from White et al.^1^ For assessing the lesions in follow-up group, the reviewers were aware of the location of treated PAVMs, and could refer to pre-treatment contrast-enhanced CT, time-resolved contrast-enhanced MRA, and any time-SLIP MRA images that were performed before embolization. However, they were blinded to the current state of the PAVMs (patent versus occluded) and to the results of post-treatment DSA, time-resolved contrast-enhanced MRA, and contrast-enhanced CT examinations. Reperfusion of PAVMs was defined on time-SLIP MRA as visualization of an aneurysmal sac corresponding to treated lesions on the time-SLIP image, because the signals of the feeding artery were disrupted by artifact susceptibility secondary to the presence of the metallic coil. On the other hand, disappearance of the aneurysmal sac was interpreted as occlusion of the PAVM. The two reviewers also evaluated reference images for each patient under consensus during a separate session. The criteria for PAVM reperfusion were <30% shrinkage of the aneurysmal sac on contrast-enhanced CT and simultaneous enhancement of the feeding artery, aneurysmal sac, and draining vein, or enhancement of the aneurysmal sac in the pulmonary arterial phase on time-resolved contrast-enhanced MRA. The criteria for complete occlusion of PAVM were marked (<30%) reduction or disappearance of the aneurysmal sac on contrast-enhanced CT and disappearance of the aneurysmal sac and draining vein in the pulmonary arterial phase on time-resolved contrast-enhanced MRA. As mentioned in previous section, although a definite diagnosis was made by DSA for both initial diagnosis and follow-up lesions, in cases where complete occlusion of PAVMs could be confirmed on both time-resolved contrast-enhanced MRA and contrast-enhanced CT, these modalities could also be used as reference-standard imaging.

### Statistical analysis

Statistical analyses were performed using commercially available software (SPSS ver. 16.0, SPSS Inc., Chicago, IL, USA). The sensitivity, specificity, and accuracy of time-SLIP MRA for the detection of reperfusion of PAVMs were calculated. Weighted κ values were used to determine interobserver agreement for image quality, location, classification, and reperfusion of PAVM on time-SLIP MRA. Interobserver agreement was scored as slight (κ < 0.21), fair (κ = 0.21 to 0.40), moderate (κ = 0.41 to 0.60), substantial (κ = 0.61 to 0.80), and almost perfect (κ = 0.81 to 1.00).

## Results

### Initial diagnosis group

**Fig. 2.**
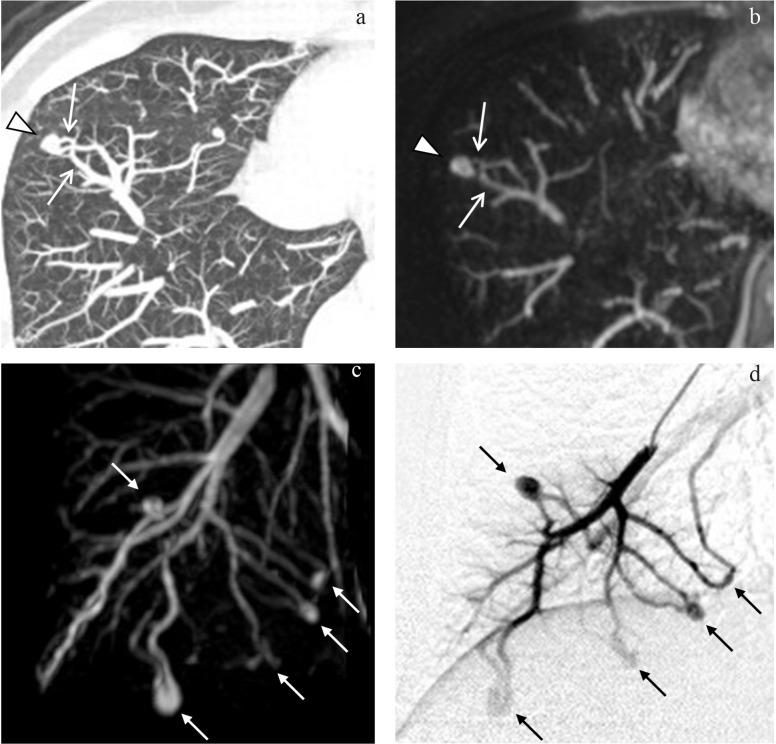
A 22-year-old woman with hereditary hemorrhagic telangiectasia who had multiple pulmonary arteriovenous malformations (PAVMs) in both lungs. (**a**) Computed tomography (CT) scan (thin-slab maximum intensity projection [MIP]) showed a simple type PAVM in the right lobe (segment 8). Arrows and arrowhead indicate the feeding artery and aneurysmal sac, respectively. (**b**) On non-contrast-enhanced magnetic resonance angiography with time-spatial labeling inversion pulse (time-SLIP MRA), the feeding artery and aneurysmal sac of the PAVM are clearly visualized, corresponding to those on the CT scan. (**c**) The MIP image of the time-SLIP MRA in the right anterior oblique view. (**d**) Multiple simple type PAVMs (arrows) originating from the same segmental artery are seen, which is well in agreement with the findings of digital subtraction angiography (DSA).

PAVM demographics and the results of image assessment are summarized in Table 1. All patients underwent time-SLIP MRA without any difficulty. In 34 (97.1%) of 35 untreated PAVMs, the image quality of time-SLIP MRA data was considered as diagnostic (image score ≥ 3). One patient with one lesion (patient 5) showed unsatisfactory image quality (image score = 2) for diagnosis of PAVM. In this case, the continuation of feeding artery and aneurysmal sac could not be visualized on either tag-off or time-SLIP images, although the detection of aneurysmal sac itself was possible. The median image quality was 4 (interquartile range, 3–4). Out of the 34 PAVMs with an image of ≥3, simultaneous visualization of the feeding artery, aneurysmal sac, and draining vein without normal pulmonary veins on the time-SLIP image was observed in 23 untreated PAVMs of three patients (patients 2, 9, and 11) whereas only the feeding artery and aneurysmal sac were simultaneously noted in other 11 lesions of seven patients (patients 1, 3, 4, 6, 7, 8, and 10). A typical image (from patient 4) is shown in Fig. 2, in which continuity of parent arteries, feeding arteries, aneurysmal sacs, and draining veins are accurately depicted. The minimum diameters detectable feeding arteries and aneurysmal sacs on time-SLIP MRA were 0.9 mm and 1.8 mm, respectively. The interobserver agreement for image quality was perfect, with a weighted κ value of 0.85. In addition, both readers were able to correctly localize and classify the PAVMs on the time-SLIP MRA images for lesions with an image quality of ≥3. The κ coefficient for both was 1.00.

### Follow-up group

**Fig. 3.**
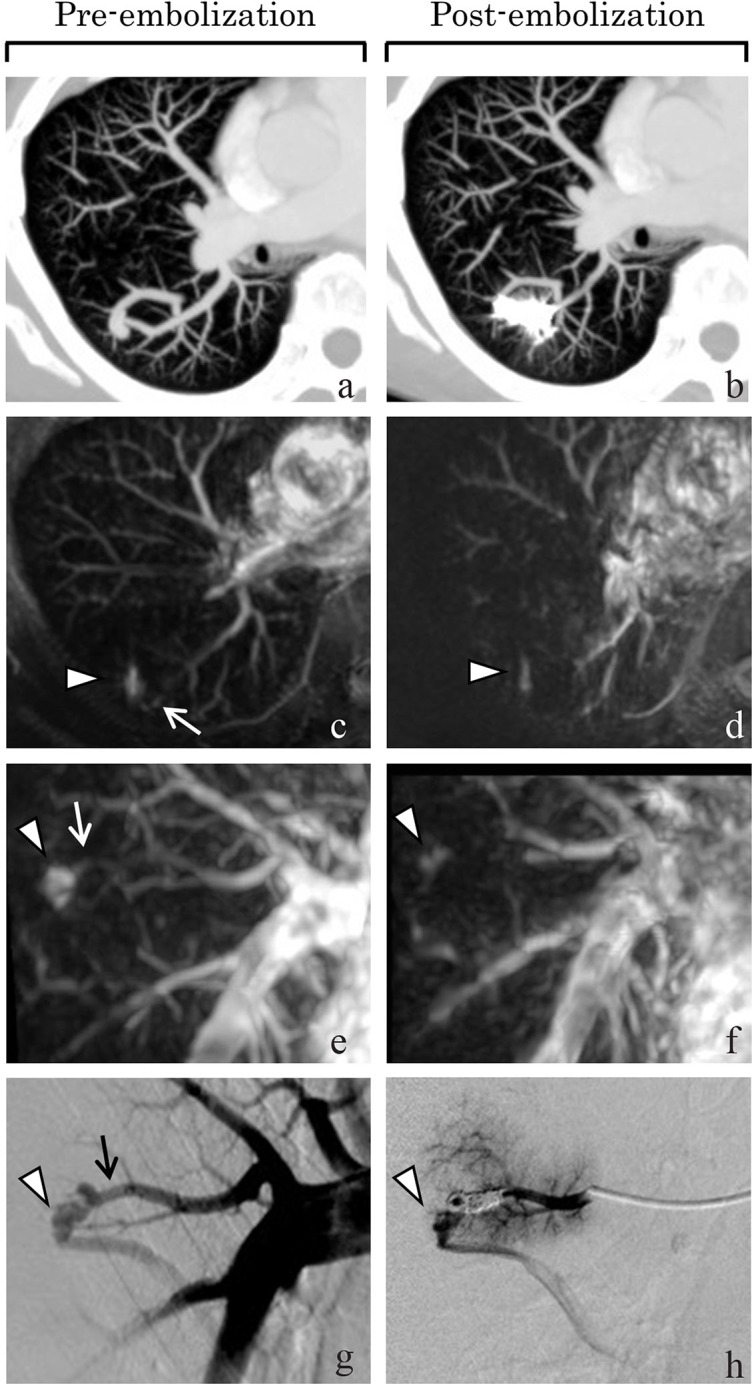
A 53-year-old man with a solitary simple type pulmonary arteriovenous malformation (PAVM) in the right upper lobe. Arrows and arrowheads indicate the feeding artery and aneurysmal sac, respectively. On contrast-enhanced computed tomography (CECT) (thin-slab maximum intensity projection [MIP]) (**a**), non-contrast-enhanced magnetic resonance angiography with time-spatial labeling inversion pulse (time-SLIP MRA) (**c**: axial image, **e**: coronal image), and digital subtraction angiography (DSA) (**g**) before embolization, a simple type PAVM connecting the segmental artery of upper lobe is seen. On CECT obtained at 6 months after embolization, the size and contrast effect of the aneurysmal sac is unclear because of prominent streak artifacts (**b**). On time-SLIP MRA, the aneurysmal sac appears at the same site as before embolization (**d**: axial image, **f**: coronal image). Recanalization was confirmed by DSA (**h**).

Time-SLIP MRA findings and results confirmed by reference standard images are summarized in Table 2. Five (17.9%) of the 28 treated PAVMs showed residue of the aneurysmal sac on time-SLIP image and the reperfusion of PAVM was confirmed by DSA in all these lesions. In all lesions with reperfusion, the cause was recanalization through coils placed in the feeding artery. In another 23 (82.1%) of 28 treated PAVMs, the aneurysmal sacs were not observed on time-SLIP images. Reference standard images showed complete occlusion in all corresponding PAVMs. The sensitivity, specificity, and accuracy for the diagnosis of PAVM reperfusion by time-SLIP MRA were all 100%. The interobserver agreement was perfect, with a weighted κ value of 1.00. Figure 3 shows a case of reperfusion of PAVM that was correctly diagnosed by time-SLIP MRA. The finding was finally confirmed by DSA.

## Discussion

To our knowledge, this is the first clinical study to evaluate the feasibility of non-contrast-enhanced MRA for assessment of PAVMs.

We first demonstrated high diagnostic performance of time-SLIP MRA for the initial diagnosis of PAVMs. Our results showed that time-SLIP MRA was able to provide selective visualization of a pulmonary-arterial dominant image corresponding to the feeding artery and aneurysmal sac comparable to DSA. In several cases, time-SLIP MRA also achieved continuous and simultaneous visualization of the feeding artery, aneurysmal sac, and draining vein of PAVMs. These findings suggest that our method can provide useful diagnostic hemodynamic information noninvasively with a capability similar to that of DSA. With our method, we also achieved a high diagnostic rate of PAVMs and a high spatial resolution in order to detect small PAVMs with feeding artery and aneurysmal sac diameters of 0.9 mm and 1.8 mm, respectively. Since the reported primary anatomical indication for embolotherapy of PAVM is a feeding artery diameter of ≥3 mm,^4,29^ we consider our method to be capable of detecting a lesion that is a candidate for embolotherapy. Further, time-SLIP MRA can provide accurate data on the vascular structure of PAVM, including branching of the feeding artery, location, and classification, which were required for developing a treatment plan. Taken together, these results indicate that time-SLIP MRA is a technically and clinically feasible modality for the initial diagnosis of PAVM. Only one lesion in one patient could not be diagnosed due to image degradation. This image degradation may have been related to the patient’s irregular breathing because our technique uses both respiratory-triggered image acquisition and a subtraction method. This is one of the drawbacks of our method, but applying a more accurate respiratory gating system, such as navigator echo-based real-time respiratory gating, may help to prevent this type of image degradation.

In the present study, the diagnostic performance of time-SLIP MRA for detection of reperfusion of embolized PAVMs was also analyzed. To assess the PAVM patency after embolization for detection of lesion of reperfusion is crucial because some patients with reperfused PAVMs develop neurologic events similar to those associated with untreated lesions.^11,12^ Although DSA is the most reliable modality to detect blood flow through the embolized lesions, it is now commonly used solely for pre-interventional evaluation during embolization therapy because of its invasiveness.^30^ The reported sensitivity and specificity of other noninvasive modalities, such as time-resolved contrast-enhanced MRA and non-contrast-enhanced CT for reperfusion, are 93% and 100% and 93% and 53%, respectively.^31^ In this study, time-SLIP MRA was able to detect reperfusion of PAVMs after coil embolization in the follow-up group with high sensitivity and specificity (both 100%), and moreover, with perfect interobserver agreement (κ = 1.00). Although we have not conducted a comparative study between these modalities, we believe that the ability of time-SLIP MRA for detection of recanalization of PAVMs after embolotherapy is equal to or greater than that of time-resolved contrast-enhanced MRA and non-contrast-enhanced CT. Our method has several advantages compared to contrast or non-contrast enhanced CT and contrast-enhanced MRA, which may be responsible for the high diagnostic accuracy and perfect interobserver agreement of time-SLIP MRA for diagnosing the reperfusion of PAVMs. First, time-SLIP MRA can show pulmonary arterial dominant images similar to those of DSA, which allow us to avoid misdiagnosing pseudo-reperfusion caused by retrograde enhancement of the venous sac originating from the collateral or draining veins that may be mistaken for true reperfusion on contrast-enhanced CT or MRA.^10,17^ Second, image distortion due to metallic artifact of time-SLIP MRA was relatively limited compared with that of CT; therefore, this method provides morphological and hemodynamic information that is more precise around the embolic materials. Third, measurement errors upon morphological evaluation with CT may be a problem, particularly for small lesions, while the diagnostic criteria for time-SLIP MRA are simpler and evaluation is easier because there is a similarity in appearance to DSA. In light of these findings, we consider time-SLIP MRA to be a promising tool for the follow-up examination of PAVMs, which may have the potential to replace currently used modalities, including CT and contrast-enhanced MRA.

Despite these advantages, there are some drawbacks of time-SLIP MRA in comparison to contrast-enhanced MRA and CT. First the acquisition time of the sequence described in this study is approximately 8 to 12 min, which is much longer than that of contrast-enhanced MRA.^17–20^ However, the longer image acquisition time improves spatial resolution, and it also reduces motion artifacts arising from the pulse wave and breathing. Additionally, whereas only one data acquisition can be performed in contrast-enhanced MRA, there is no limitation in our approach. Second, time-SLIP MRA does have limits to the number of acquisitions in a single session, and therefore it can be difficult to cover the entire lung field with high spatial resolution during a single session. The maximum acquisition range in a single session is 120 mm if the slice thickness is set to 2 mm. However, because there is no time constraint with time-SLIP MRA, the image of entire lung field can be acquired by planning multiple sessions as time allows. A third drawback of our method is that it theoretically cannot eliminate visualization of a venous sac associated with bronchial or non-bronchial systemic artery-to-pulmonary artery anastomosis, although there was no case with this type of reperfusion in the present study because the tag pulse in our method was applied to both right-sided and left-sided outflow tracts. Although this type of reperfusion is rare,^15^ optimization of the technique is required to acquire more selective visualization of pulmonary artery. Fourth, at the present stage of development of time-SLIP MRA, CT would still be necessary to develop a treatment plan for embolotherapy because the spatial resolution of the time-SLIP MRA technique is relatively low compared with that of CT, owing to susceptibility to artifacts and limitations of slice thickness imposed by the available equipment. This drawback may be avoidable with use of higher magnetic-field MR imaging scanners that can achieve higher signal-to-noise ratios.

Our study has several limitations. First, the sample size was relatively small. Second, there might be a potential selection bias because four lesions were omitted due to the limitation of acquired range in a single MR imaging session. Third, in the follow-up group, reperfusion was not confirmed by DSA in all cases. Therefore, the rate of reperfusion in our study might have been underestimated, although we believed that this possibility was low because both the contrast-enhanced CT and time-resolved contrast-enhanced MRA showed evidence of complete occlusion. Fourth, in our study, the lesions treated by sac filling or other embolic materials, including detachable balloon and vascular plugs, were not included. Therefore, further investigation to clarify the ability of time-SLIP MRA to detect these lesions would be warranted.

In conclusion, although this study was a small series and further investigation should be planned, our initial results indicate that time-SLIP MRA could be a feasible method for diagnosis and post-embolotherapy follow-up in patients with PAVM.

## Figures and Tables

**Table 1.** Pulmonary arteriovenous malformation (PAVM) demographics and summary of the diagnostic performance of non-contrast-enhanced magnetic resonance angiography with time-spatial labeling inversion pulse (time-SLIP MRA) in the initial diagnosis group

Patient no.	Lesion no.	Age (years)	Sex	PAVM demographics*				Time-SLIP MRA						DSA		

				Location	FA (mm)	AS (mm)	Status	Image quality		Location		Classification		Diagnosis	Location	Classification

								RD 1	RD 2	RD 1	RD 2	RD 1	RD 2			
1	1	53	m	r S6	2.5	7.9	untreated	4	4	r S6	r S6	simple	simple	positive	r S6	simple
2	2	70	f	r S7	4.9	10.0	untreated	3	3	r S7	r S7	simple	simple	positive	r S7	simple
3	3	46	f	l S8	5.4	8.9	untreated	4	4	l S8	l S8	simple	simple	positive	l S8	simple
4	4	47	f	r S8	1.9	4.7	untreated	3	4	r S8	r S8	simple	simple	positive	r S8	simple
5	5	65	f	r S1	2.5	11.0	untreated	2	2	ND	ND	ND	ND	positive	r S1	simple
6	6	46	m	l S4	NA	NA	treated	NA	NA	l S4	l S4	NA	NA	NA	l S4	NA
	7			r S8	2.5	9.5	untreated	3	3	r S8	r S8	simple	simple	positive	r S8	simple
7	8	62	f	r S4	3.5	11.9	untreated	3	3	r S4	r S4	simple	simple	positive	r S4	simple
8	9	81	f	r S9	5.0	27.0	untreated	4	4	r S9	r S9	simple	simple	positive	r S9	simple
	10			l S8	5.1	8.3	untreated	4	3	l S8	l S8	simple	simple	positive	l S8	simple
	11			l S9	2.9	6.5	untreated	4	4	l S9	l S9	simple	simple	positive	l S9	simple
9	12	22	f	r S5	1.6	5.4	untreated	4	4	r S5	r S5	simple	simple	positive	r S5	simple
	13			r S5	1.6	5.8	untreated	4	4	r S5	r S5	simple	simple	positive	r S5	simple
	14			r S7	NA	NA	treated	NA	NA	r S7	r S7	NA	NA	NA	r S7	NA
	15			r S8	2.0	4.5	untreated	4	4	r S8	r S8	simple	simple	positive	r S8	simple
	16			r S8	1.5	6.1	untreated	4	4	r S8	r S8	simple	simple	positive	r S8	simple
	17			r S8	1.2	2.4	untreated	4	4	r S8	r S8	simple	simple	positive	r S8	simple
	18			r S8	1.2	2.2	untreated	4	4	r S8	r S8	simple	simple	positive	r S8	simple
	19			r S8	1.3	1.8	untreated	4	4	r S8	r S8	simple	simple	positive	r S8	simple
	20			r S9	2.1	4.3	untreated	4	4	r S9	r S9	simple	simple	positive	r S9	simple
	21			l S4	2.4	5.9	untreated	4	4	l S4	l S4	simple	simple	positive	l S4	simple
	22			l S5	2.0	11.0	untreated	4	4	l S5	l S5	complex	complex	positive	l S5	complex
	23			l S5	4.0	8.9	untreated	4	4	l S5	l S5	simple	simple	positive	l S5	simple
	24			l S7	NA	NA	treated	NA	NA	l S7	l S7	NA	NA	NA	l S7	NA
	25			l S8	2.8	7.8	untreated	4	4	l S8	l S8	complex	complex	positive	l S8	complex
	26			l S8	2.0	6.7	untreated	4	4	l S8	l S8	complex	complex	positive	l S8	complex
	27			l S8	1.2	5.2	untreated	4	4	l S8	l S8	simple	simple	positive	l S8	simple
	28			l S9	1.5	4.2	untreated	4	4	l S9	l S9	simple	simple	positive	l S9	simple
	29			l S10	0.9	3.5	untreated	4	4	l S10	l S10	simple	simple	positive	l S10	simple
	30			l S10	1.2	4.3	untreated	4	4	l S10	l S10	simple	simple	positive	l S10	simple
10	31	65	f	r S5	4.9	11.2	untreated	4	4	r S5	r S5	simple	simple	positive	r S5	simple
	32			r S7	4.5	7.7	untreated	3	3	r S7	r S7	simple	simple	positive	r S7	simple
	33			l S3	3.0	5.8	untreated	3	3	l S3	l S3	simple	simple	positive	l S3	simple
11	34	44	f	r S5	2.5	5.2	untreated	3	3	r S5	r S5	simple	simple	positive	r S5	simple
	35			r S8	3.1	4.1	untreated	3	3	r S8	r S8	simple	simple	positive	r S8	simple
	36			r S9	3.8	15.1	untreated	4	4	r S9	r S9	simple	simple	positive	r S9	simple
	37			r S9	7.5	11.2	untreated	4	4	r S9	r S9	simple	simple	positive	r S9	simple
	38			l S10	5.6	8.5	untreated	4	4	l S10	l S10	simple	simple	positive	l S10	simple

Average					2.9	7.5	Weighted κ value	0.85		1.00		1.00				
Median		53			2.5	6.5										
Range		22–81			0.9–5.6	1.8–27.0										

* Evaluated on computed tomography; AS, diameter of aneurysmal sac; DSA, digital subtraction angiography; f, female; FA, diameter of feeding artery; l, left; m, male; NA, not applicable; ND, not determined; r, right; RD, reader

**Table 2.** Summary of the diagnostic performance of non-contrast-enhanced magnetic resonance angiography with time-spatial labeling inversion pulse (time-SLIP-MRA) in the follow-up group

Patient no.	Lesion no.	Location	Method of embolization	Reference standard imaging	Time-SLIP MRA		Reference standard	

					Reperfusion		Reperfusion	Type

					RD 1	RD 2		
1	1 pe	r S6	MC/FA	DSA	positive	positive	positive	recanalization
2	2 pe	r S7	MC/FA	DSA	positive	positive	positive	recanalization
3	3 pe	l S8	MC/FA	TR-CEMRA, CECT	negative	negative	negative	NA
4	4 pe	r S8	MC/FA	TR-CEMRA, CECT	negative	negative	negative	NA
6	6^†^	l S4	MC/FA	DSA	positive	positive	positive	recanalization
	7 pe	r S8	MC/FA	DSA	negative	negative	negative	NA
8	9 pe	r S9	MC/FA	DSA	negative	negative	negative	NA
	10 pe	l S8	MC/FA	TR-CEMRA, CECT	negative	negative	negative	NA
	11 pe	l S9	MC/FA	TR-CEMRA, CECT	negative	negative	negative	NA
9	12 pe	r S5	MC/FA	DSA	negative	negative	negative	NA
	13 pe	r S5	MC/FA	DSA	negative	negative	negative	NA
	14^†^	r S7	MC/FA	DSA	positive	positive	positive	recanalization
	15 pe	r S8	MC/FA	DSA	negative	negative	negative	NA
	16 pe	r S8	MC/FA	DSA	negative	negative	negative	NA
	17 pe	r S8	MC/FA	DSA	negative	negative	negative	NA
	18 pe	r S8	MC/FA	DSA	negative	negative	negative	NA
	19 pe	r S8	MC/FA	DSA	negative	negative	negative	NA
	20 pe	r S9	MC/FA	DSA	negative	negative	negative	NA
	22 pe	l S5	MC/FA	DSA	negative	negative	negative	NA
	24^†^	l S4	MC/FA	DSA	positive	positive	positive	recanalization
	25 pe	l S8	MC/FA	DSA	negative	negative	negative	NA
	26 pe	l S8	MC/FA	DSA	negative	negative	negative	NA
	27 pe	l S8	MC/FA	DSA	negative	negative	negative	NA
	28 pe	l S9	MC/FA	DSA	negative	negative	negative	NA
11	34 pe	r S5	MC/FA	DSA	negative	negative	negative	NA
	35 pe	r S8	MC/FA	DSA	negative	negative	negative	NA
	36 pe	r S9	MC/FA	DSA	negative	negative	negative	NA
	37 pe	r S9	MC/FA	DSA	negative	negative	negative	NA

Weighted κ value 1.00								
Sensitivity (%)100								
Specificity (%)100								
Diagnostic accuracy (%)100								

† , treated lesion at initial time-SLIP MRA; CECT, contrast-enhanced computed tomography; DSA, digital subtraction angiography; FA, embolization of feeding artery; l, left; MC, metallic coil; NA, not applicable; pe, post-embolization; r, right; RD, reader; TR-CEMRA, time-resolved contrast-enhanced magnetic resonance angiography
